# The Factor Structure, Predictors, and Percentile Norms of the Center for Epidemiologic Studies Depression (CES-D) Scale in the Dutch-speaking Adult Population of Belgium

**DOI:** 10.5334/pb.261

**Published:** 2016-01-18

**Authors:** Qian Wu, Yasemin Erbas, Annette Brose, Peter Kuppens, Rianne Janssen

**Affiliations:** 1 Faculty of Psychology and Educational Sciences, KU Leuven, Belgium

**Keywords:** depression, the CES-D, second-order factor, MIMIC, percentile norms

## Abstract

The Center of Epidemiologic Studies Depression Scale (CES-D) is a commonly used self-report scale to measure depressive symptoms in the general population. In the present study, the Dutch version of the CES-D was administered to a sample of 837 Dutch-speaking adults of Belgium to examine the factor structure of the scale. Using confirmatory factory analysis (CFA), four first-order models and two second-order models were tested, and the second-order factor model with three pairs of correlated error terms provided the best fit to the data. Second, five socio-demographic variables (age, gender, education level, relation status, and family history of depression) were included as covariates to the second-order factor model to explore the associations between background characteristics and the latent factor depression using a multiple indicators and multiple causes (MIMIC) approach. Age had a significantly negative effect on depression, but the effect was not substantial. Female gender, lower education level, being single or widowed, and having a family history of depression were found to be significant predictors of higher levels of depression symptomatology. Finally, percentile norms on the CES-D raw scores were provided for subgroups of gender by education level for the general Dutch-speaking adult population of Belgium.

Depression is one of the leading causes of disability, affecting a large number of people all over the world (WHO, 2012). *The Fifth Edition of the Diagnostic and Statistical Manual of Mental Disorder* (DSM-5, 2013) defines major depressive disorder (MDD) as a mental disorder characterized by symptoms of depressed mood, loss of interests or pleasure, appetite change, sleep disturbance, psychomotor changes, feelings of worthlessness or guilt, and recurrent suicidal thoughts. Among these symptoms, depressed mood and loss of interests or pleasure are regarded as two key dimensions of MDD (Vares, Salum, Spanemberg, Caldieraro, & Fleck, 2015; Vrieze et al., 2014). While the DSM-5 treats depression as a psychological disorder, many self-report scales of depression consider it as a syndrome (Schroevers, Sanderman, Van Sonderen, & Ranchor, 2000), and thus, they assess depression by measuring a group of depressive symptoms in individuals to facilitate the screening and early diagnosis of depression.

The Center for Epidemiologic Studies Depression Scale (CES-D; Radloff, 1977) is one of the most commonly used self-report depression scales to measure depressive symptomatology in the general population. The scale consists of 20 items selected from previously validated depression scales. Each item measures one core component of depressive symptoms, and together the whole scale is considered to provide a valid and reliable measurement of the multidimensional construct of depression. Ever since its publication, the CES-D has been shown to have high internal consistency and well established validity in both clinical and community-based populations in diverse contexts across cultures (e.g., Beekman et al., 1997; Barkmann, Erhart, Schulte-Markwort, & the BELLA Study Group, 2008; Gonçalves & Fagulha, 2004; Hann, Winter, & Jacosen, 1999; Schroevers et al., 2000; B. Zhang et al., 2011; W. Zhang et al., 2012).

Using principal component analysis (PCA), Radloff (1977; 1991) identified that four factors could be extracted from the intercorrelations among the CES-D items, which were Depressed Affect, (absence of) Positive Affect, Somatic Symptoms, and Interpersonal Relations. This four-factor structure has been generally supported and replicated in subsequent studies, and further substantiated by meta-analytic evidence on the basis of 28 studies (Schafer, 2006). However, alternative factor structures have also been hypothesized and examined in recent factor analytic studies. For example, a two-factor solution posits all Positive Affect items as one factor and the remaining items as the other factor of general negative affect (Schroevers et al., 2000). Another frequently reported model is a three-factor solution combining Depressed Affect and Somatic Symptoms into one factor and retaining Positive Affect and Interpersonal Relations as the other two (Guarnaccia, Angel, & Worobey, 1989; B. Zhang et al., 2011). Further, a higher-order factor model postulates a second-order factor underlying the original four factors by Radloff (Gonçalves & Fagulha, 2004; Morin et al., 2011; Sheehan, Fifield, Reisine, & Tennen, 1995). All those factor models were found plausible and provided satisfactory model fit to the data in the respective studies.

## The Present Study

Compared to international standards, the CES-D has not often been implemented and investigated in depression research in the Belgian context. Previous studies on the Dutch version of the scale have been limited either to abridged versions of the scale, like the CES-D 8 (Van de Velde, Levecque, & Bracke, 2009) and the CES-D 10 (Schroevers et al., 2000), or restricted to samples of elder people (B. Zhang et al., 2011). To our knowledge, the complete Dutch version of the 20-item scale has not been studied in a large general population of Belgium. Therefore, in the present study the CES-D is administered to a sample of Dutch-speaking adults of Belgium. The purpose of the study is threefold: (a) to investigate the factor-structure of the Dutch version of the CES-D; (b) to explore the associations of depression with socio-demographic characteristics; (c) to collect normative data of Dutch-speaking Belgian population on the CES-D.

The first objective of the current study is to examine the factor structure of the CES-D in a sample of Dutch-speaking Belgian adults. Given the various models proposed in previous studies, we will focus and compare six models that are mostly considered. Those models will be examined using confirmatory factor analysis (CFA), as it provides a more compelling analytic framework than exploratory factor analysis like PCA in terms of psychometric evaluation and construct validation (Brown, 2006). Moreover, responses to the CES-D items are measured on a four-point scale, which are categorical and highly skewed. This ordered categorical nature and non-normality of response data will be taken into account by using the robust weighted least square (WLSMV) estimation instead of maximum likelihood (ML) estimation, as the latter can produce incorrect parameter estimates, standard errors, and test statistics with such categorical data (Brown, 2006).

The second objective of this study is to evaluate associations between depression and several socio-demographic characteristics. The risk of developing depression is known to be related to a number of background factors. Studies showed that old people are at higher risk of developing depression (Patel et al., 2009; WHO, 2012). However, other studies reported a negative relation between age and depression (Bromet, 2011; Offord et al., 1996, Streiner, Cairney, & Veldhuizen, 2006). Gender is also a risk factor for depression, with women being two or three times more likely to suffer from depression than men (e.g., Bromet et al., 2011; Rai et al., 2013; WHO, 2012). Other factors associated with a higher risk of depression are social and economic disadvantages, such as poverty (Patel et al., 2009), low education (Akhtar-Danesh & Landeen, 2007; Bjelland et al., 2008), a family history of depression (Levinson, 2006; Monroe, Slavich, & Gotlib, 2014), and environmental factors (Polanczyk et al, 2009). In the present study, associations between depression and those factors will be investigated using a multiple indicators and multiple causes (MIMIC) approach, which is a special case of structural equation modeling where latent variables (and indicators) are regressed on covariates to explore the relationship between the covariates and the measurement model (Buehn & Schneider, 2008).

Finally, the third objective of this study is to provide normative data on the CES-D. Few studies had the attempt to provide normative data of the CES-D for general population. In the literature, only Crawford et al. (2003; 2009; 2011) did a series of studies to present percentile norms of the CES-D for UK and Australian adult population. In this study, percentile norms of the CES-D for the Dutch-speaking adult population of Belgium are presented.

## Method

### Participants

A sample of 837 people was drawn from the adult population of Flanders, the Dutch-speaking region of Belgium. Participants were recruited by students following the course of psychometrics at the KU Leuven. The sampling procedure was done in the following way. First, to facilitate sampling and ensure a representative sample, the Flemish adult population was stratified using three variables age, gender, and education level. Age varied between 18 and 74, and was classified into six groups (see Table 1). Education level was dichotomized into lower and higher on the basis of whether or not a person had a degree in post-secondary education. This led to 24 different profiles, and the initial number of participants of each profile was in line with the relative occurrence of the profile in the Flemish population (Algemene Directie Statistiek en Economische Informatie, ADSEI, 2012). Second, each student was responsible to find three participants, each matching one of these profiles. Table 1 shows the percentages of the recruited sample along the three stratification variables. Note that this table was based on 821 participants, as 16 participants filled in an age that was invalid or out of the intended age range. The distribution of gender in the sample corresponded to the sex ratio in the general Flemish population (*χ*^2^ = 7.61, *p* = .06). However, the distributions of the sample over age groups ( *χ*^2^ = 17.33, *p* = .00) and education level (*χ*^2^ = 15.59, *p* = .00) indicated that they significantly differed from the distributions in the population. The subgroup of participants in the 55–64 age group with lower education level was found less represented in the sample, contributing to the large value of the *χ*^2^.

**Table 1:** Percentage of Distribution of Age, Gender, and Education Level in the Sample (n = 821).

Gender	Education Level	Age						Total

		18–24	25–34	35–44	45–54	55–64	65–74	

Female	Low	3.90	3.90	4.75	7.80	4.26	5.48	30.09
	High	2.68	5.12	5.24	3.78	2.80	1.58	21.19
Male	Low	4.38	5.72	4.14	7.92	4.75	3.29	30.21
	High	2.31	4.14	3.78	4.14	2.68	1.46	18.51
Total		13.28	18.88	17.90	23.63	14.49	11.81	100.00

### Measurements

#### The CES-D

The Dutch translation of the CES-D by Bouma et al. (1995) was used to measure depressive symptoms among participants. Of the 20 items, 16 measure negative feelings, such as “I felt sad”, and four mirror items measure positive affect, such as “I was happy”. Respondents were asked to indicate the frequency of occurrence of the symptoms or behaviors mentioned in the items during the past week on a four-point scale: (0) rarely or none of the time (less than 1 day); (1) some or a little of the time (1–2 days); (2) occasionally or a moderate amount of time (3–4 days); (3) most or all of the time (3–7 days). The common scoring of the CES-D is the sum of 20 items, with four mirror items reverse-coded. The total score ranges from 0 to 60, with higher scores indicating more depressive symptoms and higher frequencies of experiencing those symptoms.

#### Socio-demographic Characteristics

A demographic questionnaire was used to collect background characteristics of the participants, including age, gender, education level, and relationship status (single, in relationship but non-cohabiting, married/in relationship and co-habiting, and widowed). The family history of depression was probed as part of a family history of mental illness questionnaire. Respondents were required to specify whether they or their family member(s) had ever suffered from any mental disorders, including MDD, bipolar disorder, schizophrenia, autism, and borderline personality disorder. In the present study, only the results regarding MDD were used, with 1 indicating having a family history of depression, and 0 no family history of depression.

### Procedure

Data were collected through an online survey, which participants completed at home within a five-day time window. The online survey consisted of six questionnaires, including demographic questionnaire, family history of mental illness, the CES-D, and three other questionnaires which were not related to the present research questions. The whole online survey lasted approximately one hour in total, but participants were allowed to complete the survey at different times with breaks in between.

### Ethics

This study was approved by the ethical commission of the Faculty of Psychology and Educational Sciences at the KU Leuven. Participants provided the Informed Consent before proceeding to participating in the study.

### Data Analysis

CFA was used to examine possible factor structure models underlying the CES-D. Six competing models were tested: (1) a one-factor model; (2) a two-factor model with all positive affect items loading on one factor and the remaining items of negative feelings loading on the other one; (3) a three-factor model combining Radloff’s Depressed Affect and Somatic Symptoms into one factor, and Positive Affect and Interpersonal Relations as the other two; (4) the four-factor model proposed by Radloff; (5a) a second-order factor model with a single higher-order factor underlying the original four factors; (5b) the same second-order factor model with correlated errors between three pairs of items.

As responses to the CES-D items were measured on a four-point scale, categorical outcomes are better approached with robust weighted least square (WLSMV) estimation using polychoric correlation matrices (Brown, 2006). Because chi-square tests are sensitive to sample size, it frequently leads to model rejection with large samples, and therefore the goodness-of-fit of competing models was evaluated along the following criteria: root mean square error of approximation (RMSEA < 0.08), comparative fit index (CFI > 0.95), and Tucker-Lewis index (TLI > 0.90) (Hu & Bentler, 1999). The modification indices (MI) and expected parameter changes (EPC) were used to identify focal areas of misfit and provide possible model improvement. Then the best fitting model was retained and used in subsequent analyses.

To examine the effect of socio-demographic variables on depression, a MIMIC approach was applied by incorporating age, gender (female coded as 0, and male 1), education level, relation status (regrouped into two categories with 0 indicating being single/widowed, and 1 in a relationship/married), and family history of depression as covariates in the best fitting model. Each covariate was specified a direct path to the latent factor(s). A regression coefficient significantly different from zero indicated a significant direct effect of covariates on the latent factor(s), and also implied different latent means at different levels (groups) of covariates.

Finally, the raw scores of the CES-D were converted into percentiles separately for demographic variables that turned out to be significant risk factors for depression.

All analyses were conducted using the R package “lavaan” (Rosseel, 2012).

## Results

### Factor Structure of the CES-D

Table 2 presents the summary of goodness-of-fit of six competing models of the CES-D. Model 1 tested the one-factor model, expressing the hypothesis that responses to the CES-D items can be explained by a single underlying factor. The large *χ*^2^ value and that the three fit indices RMSEA, CFI, and TLI did not reach the criteria indicated a rather poor fit of this model. Model 2, 3, and 4 specified a model with two, three, and four factors, respectively. Compared to Model 1, these three models provided a better fit to the data with substantial drops in the *χ*^2^ value and all fit indices meeting the evaluation criteria. The results also suggested that the four-factor model proposed by Radloff (Model 4) had the best model fit among the first four competing models.

**Table 2:** Summary of the Goodness-of-Fit Indices of the CES-D Models.

Model		*χ*^2^	*df*	RMSEA	90% CI	CFI	TLI

1.	One-factor	1360.029	170	0.092	(0.087, 0.096)	0.882	0.868
2.	Two-factor	690.940	169	0.061	(0.056, 0.066)	0.948	0.942
3.	Three-factor	559.522	167	0.053	(0.048, 0.058)	0.961	0.956
4.	Four-factor	472.141	164	0.047	(0.042, 0.052)	0.969	0.965
5a.	Second-order	490.122	166	0.048	(0.043, 0.053)	0.968	0.963
5b.	Second-order with correlated errors	339.865	163	0.036	(0.031, 0.041)	0.982	0.980

*Note.* CI = Confidence interval.

**Figure 1:**
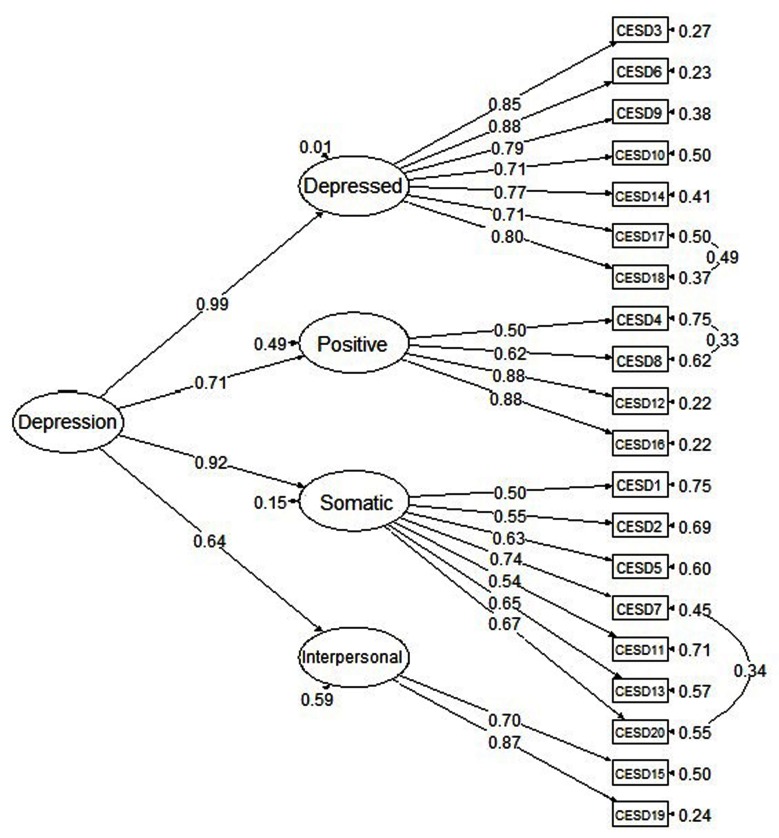
The second-order factor model with correlated errors of the CES-D (Model 5b) and completely standardized parameter estimates.

In Model 4, the four factors were shown to be highly correlated with correlations ranging from 0.47 to 0.88 (*p* < .001). This suggested the possibility of a higher-order factor that can account for these strong correlations among the four factors. Thus, Model 5a tested this hypothesis, and it yielded similar fit indices as Model 4. Inspections of MI and EPC indicated that Model 5a can be further improved if the constrained residual covariance was set free between Item 4 (“I felt that I was as good as other people) and Item 8 (“I felt hopeful about the future”), between Item 17 (“I had crying spells”) and Item 18 (“I felt sad”), and between Item 7 (“I felt that everything I did was an effort”) and Item 20 (“ I could not get ‘going’”). Those correlated error terms have been reported in previous studies, and were found reasonable. Hence, in Model 5b, the three sets of residual covariances were estimated freely. By allowing the correlated errors, there was a substantial improvement in the model fit of Model 5b. The model had the lowest *χ*^2^ value, and RMSEA, CFI, and TLI were also improved. The likelihood ratio test suggested that Model 5b had a significant model fit improvement over Model 5a (Δ*χ*^2^ = 125.67, Δ*df* = 3, *p* < .001). Examination of the parameter estimates showed that all factor loadings were significantly different from zero, and all four first-order factors (Depressed Affect, Positive Affect, Somatic Symptoms, and Interpersonal Relations) loaded strongly on the second-order factor (Depression), with loadings ranging from 0.64 to 0.99 (*p* < .001). The CFA results suggested that Model 5b of the second-order single factor with correlated errors had the best fit to the data, and thus, it was retained as the best fitting model for the subsequent analyses. Figure 1 presents the factor structure of Model 5b with the corresponding factor loadings and residual variances and covariances.

### MIMIC Model

Five covariates, age, gender, education level, relation status, and family history of depression were added as covariates in the best fitting model (Model 5b). Each covariate was estimated with a direct path to the second-order factor of depression. The MIMIC model yielded a satisfactory fit to the data as well (*χ*^2^ = 585.220, *df* = 258, RMSEA = 0.039, CFI = 0.970, TLI = 0.966) and did not produce large MI values of misfit, suggesting that the inclusion of the covariates did not alter the facture structure. Table 3 presents the regression coefficients of the covariates of the second-order factor depression in the MIMIC model. Age was found to be a significant predictor of depression, but its magnitude was rather small. Gender had a negative effect on depression, with men scoring on average 0.397 units lower than women. Education level also had a significant negative effect on depression, with higher educated people scoring on average 0.265 units lower than people with lower education. Relation status was also found to have a significant negative effect on depression, whereas having a family history of depression was shown to have a positive effect on the latent factor depression.

**Table 3:** Parameter Estimates and Statistics for the Covariates of Depression from the MIMIC Model.

Covariate	Estimate	S.E.	Z-value	P(>|z|)

Age	−0.006	0.002	−2.385	0.017
Gender	−0.397	0.080	−4.986	0.000
Education Level	−0.265	0.082	−3.229	0.001
Relation Status	−0.446	0.102	−4.365	0.000
Family History of Depression	0.171	0.089	1.927	0.054

### Percentile Norms

The MIMIC model results showed that gender and education level had significant effects on the factor of depression, indicating different latent means across gender and education levels. Thus, percentile norms were generated separately for these subgroups (Table 4). It can be seen that female with lower education level had higher scores on the scale than other subgroups on almost all percentile ranks.

**Table 4:** Percentiles of the CES-D Scores for Subgroups of Gender by Education Level for Dutch-Speaking Belgian Adults Population.

Gender	Education Level	Percentile								

		1	5	10	25	50	75	90	95	99

Female	Lower	0	1	3	6	11	17	25	33	41
	Higher	0	0	1	4	8	13	20	26	39
Male	Lower	0	1	1	4	7	12	19	25	34
	Higher	0	0	0	4	7	12	17	19	30

## Discussion

Despite extensive international studies and investigations of the CES-D, the examination of the scale has been comparatively limited to a subset of the items, or restricted to a specific subpopulation in the Belgian context. In the present study, the Dutch version of the CES-D was administered to a sample of Dutch-speaking Belgian adults. Given the inconsistencies of the factor structure of the scale in previous studies, we first examined its factor structure. Based on the best fitting model found in our sample, we further explored the associations between depression and several socio-demographic characteristics. Normative data were also generated for different subgroups of the general population.

Six competing factor models, including four first-order and two second-order factor models, were tested. On the one hand, in line with the majority of previous studies (e.g., Guarnaccia et al., 1989; Morin et al., 2011; Shafer, 1996; Schroevers et al., 2000; Sheehan et al., 1995; B. Zhang et al., 2011), the results in the present study confirmed that the four-factor structure suggested by Radloff (1977) provided a better model fit in comparison with other three first-order factor models. On the other hand, the four factors were highly correlated suggesting a possible higher-order factor underlying the moderate and strong correlations among these four factors. Our results confirmed this hypothesis, with a substantial improvement in model fit in the second-order factor model with correlated errors between three pairs of items (Model 5b). Inclusion of correlated error terms in cross-sectional studies was not favored by Jöreskog (1993), however, the three pairs of residual covariances included in the current study have been repeatedly reported in previous studies (e.g., Sheehan et al., 1995; Van de Velde, 2009). This recurrent finding suggests that the relationships among those items can indeed be accounted for by some external causes apart from latent factors, and thus justifies the inclusion of correlated errors. Therefore, the second-order model with correlated errors was retained as the best fitting model of the six models tested in the present study. As such, this second-order model is more informative in such a way that the CES-D items are indicators of core components of depressive symptoms and the scale as a whole measures a single underlying construct of depression from four sub-dimensions. This model is also better conceptually attuned with the recognition of depression as a multidimensional construct (DSM-5, 2013; Vares et al., 2015; Vrieze et al., 2014), and supports the plausibility of the common use of sum score of the 20 items.

The second objective of the present study was to examine the associations between socio-demographic characteristics and the latent factor of depression. Using a MIMIC model approach, age, gender, education level, relation status, and family history of depression were included as covariates in the Model 5b. In previous studies, there were no consistent results with respect to the relationship between age and depression. In our study, a significant negative relationship of age was observed, but the effect was relatively small. Gender (male) was found to have a negative effect on the latent factor of depression, which is in line with prior studies (e.g., Carleton et al., 2013; Morin et al., 2011; Yang & Jones, 2007). This gender difference on the latent factor suggests that on average women report more depressive symptoms than men do. Higher education level and relationship status were found to have a negative effect on depression. The findings are consistent with the conclusion in previous studies that higher education level has a protective effect against depression (Bjelland et al., 2008, Lorant et al., 2003; Patel et al., 2009; Rai et al., 2011), and that being separated or widowed increases the risk of having depression (Akhtar-Danesh & Landeen, 2007; Bromet et al., 2011). Our results also support previous studies which showed that people having a family history of depression were two or three times more likely to develop depression at some point in their life (Levinson, 2006; Monroe, Slavich, & Gotlib, 2014; Rai et al., 2013; WHO, 2012).

The final objective of this study was to provide normative data on the CES-D scale for the general Dutch-speaking adult Flemish population. Because of the significant effects of gender and education level on the latent factor of depression found in this study, percentile norms were generated separately for these subgroups. Lower educated females scored higher on nearly all percentiles, which reflected the significant effects of gender and education level on depression. Percentiles can function as a supplement to the traditional cut-off score(s) for the CES-D. Expressing the scale scores in percentile ranks is also in accordance with the conception of depression as a dimensional rather than categorical construct (Crawford et al., 2009; 2011).

Some limitations of the current study should be noted. First, although stratification by age, gender, and education level was done with the attempt to obtain a representative sample, the final sample recruited by students was not a fully representative of the general Dutch-speaking population of Belgium. This may have some impacts on the generalizability of our results. Second, some of the socio-demographic variables were not measured with conventional categorizations, such as education level which was only measured at lower and higher levels, and relation status which was regrouped into two categories. Specific categorizations will be needed to obtain a better understanding of the associations between background characteristics and depression. Also, in the present study, we only identified the group differences on the latent means across gender and educational levels. The equivalence of the measurement model across those subgroups was not fully examined. Based on the factor model we have established in the current study, further analyses can be performed to evaluate the measurement invariance of the complete set of the CES-D items across gender and education levels in the Dutch-speaking Belgian population.

## Competing interests

The authors declare that they have no competing interests.
